# COMP Report: Patient‐specific quality assurance practices in Canadian radiotherapy—results from a national survey

**DOI:** 10.1002/acm2.70591

**Published:** 2026-04-21

**Authors:** Muoi N. Tran, Renée‐Xavière Larouche, Brennen Dobberthien, Nicolas Varfalvy, Gerard Lagmago Kamta, Philip Wright, Richard Lee, Gordon H. Chan

**Affiliations:** ^1^ Royal Victoria Regional Health Centre Barrie Ontario Canada; ^2^ Centre hospitalier de l'Université de Montréal (CHUM) Montréal Québec Canada; ^3^ BC Cancer‐Surrey Surrey British Columbia Canada; ^4^ CIC Hôpital Enfant‐Jésus CHU de Québec ‐ Université Laval Québec Québec Canada; ^5^ CISSS de la Montérégie‐Centre – Hôpital Charles‐Le Moyne, Greenfield Park Québec Canada; ^6^ Saskatoon Cancer Centre, College of Medicine University of Saskatchewan Saskatoon Saskatchewan Canada; ^7^ CancerCare Manitoba Winnipeg Manitoba Canada; ^8^ Juravinski Cancer Centre Hamilton Health Sciences Hamilton Ontario Canada

**Keywords:** national survey, practice patterns, PSQA, SBRT, VMAT

## Abstract

Patient‐specific quality assurance (PSQA) is an essential component of modern radiotherapy, ensuring safe and accurate delivery of increasingly complex treatments. Given its resource‐intensive nature and the availability of evolving approaches, examining how PSQA is implemented and optimized across Canada is important to inform best practices. This study aimed to characterize the current landscape of PSQA practice across Canadian radiotherapy centers, identify patterns and regional variations, and explore opportunities for future national guidance. A 30‐question web‐based survey was developed and distributed in March 2024 to all Canadian radiotherapy centers, including satellites. The survey addressed staffing, treatment machines, PSQA tools and methodologies, policies, data analysis, frequency of PSQA, and failure management. Responses were collected in both English and French, de‐identified, and analyzed by center size and region (British Columbia, Prairies, Ontario, Quebec, and Atlantic). The survey achieved a 90% response rate (45 of 50 centers). Most centers reported using 3D array detectors, followed by EPID and point detectors. Gamma index analysis was the predominant evaluation method. More than half of participating centers, including 65% of large centers, reported reducing PSQA measurement frequency, citing accumulated local experience rather than external guidance. However, all centers in the Prairies and Atlantic regions reported maintaining full measurement frequency. A vast majority of centers that used delivery log calculations also reported a reduction in measurement frequency, while most centers using EPID did not. Across all regions, selective adoption of recommendations from both Canadian and American guidelines was observed. PSQA practices in Canada are heterogeneous, reflecting local resources, institutional experience, and evolving international recommendations. Given the resource‐intensive nature of PSQA and observed variability in practice, these findings highlight the value of developing national guidance tailored to Canadian clinical realities.

## 1 INTRODUCTION

Radiation therapy is an integral component in the management of malignancies, contributing significantly to cancer control and cure rates worldwide. Advances in delivery techniques, such as intensity‐modulated radiation therapy (IMRT) and volumetric modulated arc therapy (VMAT), have significantly improved dose conformity and sparing of organs at risk. However, these benefits are accompanied by increased complexity in planning and delivery, underscoring the need for robust quality assurance (QA) frameworks^1^, ^2^ and references therein.

Patient‐Specific Quality Assurance (PSQA) plays a critical role in modern radiotherapy by verifying the accuracy and deliverability of individual treatment plans. It provides a comprehensive verification of the treatment planning and delivery process, ensuring that the approved treatment plan can be executed safely and accurately on the treatment machine. In Canada, Canadian Partnership for Quality Radiotherapy (CPQR) has published technical quality control (QC) guidelines for patient‐specific dosimetric measurements to promote consistent QA standards across the country, including specific recommendations for PSQA.^3^ While these guidelines are widely known in Canada, the degree of their adoption and implementation across Canadian centers remains unclear.

Several national and regional surveys have explored PSQA practices. In Canada, Chan et al.^4^ conducted a regional assessment of PSQA implementation in Ontario across centers, identifying variations in tool usage, staffing models, and interpretation of pass/fail criteria. Pan et al.^5^ and Tassano‐Smith et al.^6^ reported on patient‐specific IMRT QA practices across China and the UK, both noting wide variability in measurement tools and thresholds. Marino et al.^7^ performed a national survey on stereotactic body radiation therapy (SBRT) PSQA practices in Italy, highlighting challenges with resource allocation and interpretation of QA metrics. Similarly, Mehrens et al.^8^ documented QA practices for 3D conformal radiotherapy and IMRT/VMAT in the United States, revealing significant heterogeneity in applying the American Association of Physicists in Medicine (AAPM) Task Group 218 (TG‐218) report recommendations.^1^ This study provides the first national survey focused on PSQA practices across all radiation treatment centers in Canada.

The goals of this study were threefold: (1) to survey and characterize existing PSQA processes and technologies; (2) to assess national patterns and highlight regional and institutional variations; and (3) to identify challenges, needs, and opportunities that could inform future development of Canadian‐centric best practice guidelines. This manuscript presents the results of the survey, providing an overview of PSQA across Canada and underscoring the need for a national dialogue to advance standardization and establish practical, consistent, and effective guidelines. It should be noted that reported practices are described to illustrate current variability and evolving approaches; they are not intended as prescriptive recommendations.

## 2 MATERIALS AND METHODS

### 2.1 National PSQA working group

A community‐driven working group on PSQA practice was formed in 2024, consisting of volunteers from National Quality Community of Practice (NQCP). NQCP, established under CPQR, serves as a national forum for sharing expertise and strengthening quality and safety in radiation therapy across Canada. The PSQA working group was comprised of ten clinical physicists and one radiation oncologist, representing cancer centers nationwide and reflecting both official language groups. Its objectives were to capture a snapshot of current practices and gauge future directions for PSQA, with a focus on routine VMAT and SBRT treatments.

### 2.2 Survey design and distribution

A comprehensive survey on PSQA practice was developed and distributed in March 2024 through the Canadian Organization of Medical Physicists to the heads of medical physics departments in all Canadian radiotherapy centers, including satellites. For this survey, PSQA was defined as either a patient‐specific measurement or a delivery log‐based calculation using machine beam parameters captured during treatment delivery. Pre‐delivery calculation and beam transfer integrity checks were excluded. The scope was restricted to routine VMAT and SBRT deliveries, excluding static IMRT and stereotactic radiosurgery. Survey questions were generated by group members based on published surveys, existing guidelines, and clinical experience. Questions were then refined and selected through group consensus. To ensure objectivity, the survey was vetted by internal reviewers not involved in question development and one external reviewer.

The final survey contained 30 multiple‐choice questions with optional free‐text comments. Topics included staffing, treatment platforms, policies and procedures, PSQA methodologies and tools, measurement devices, data analysis practices, frequency of PSQA, and failure management. Treatment platforms covered C‐arm linacs, O‐ring linacs, MR linacs, and robotic systems. Measurement devices included 2D array, 3D array, EPID, film, point, and transmission detectors. In addition, delivery log‐based dose reconstruction/calculation was included as a verification tool. Centers were asked to submit a single response, ideally completed by the physicist(s) most familiar with their PSQA program and reflecting a collective departmental view. Completion time was estimated at 30–60 minutes.

The survey was hosted on Research Electronic Data Capture software (REDCap, Vanderbilt University, Nashville, Tennessee, USA) and distributed in both English and French. Participating centers were required to identify themselves for potential follow‐up clarifications. The survey closed in April 2024.

### 2.3 Data analysis

Responses and comments were first reviewed for accuracy, consistency, and clarity. When clarification was needed, follow‐up questions were sent to individual centers, and responses were updated accordingly before the data were de‐identified and processed. The dataset was then exported to Microsoft Excel (Microsoft Corp., Redmond, WA) for analysis. Pivot tables and charts were used to summarize center characteristics, identify patterns, and explore regional variations. Data were grouped by geographical region and center size, ensuring a minimum grouped size of four to preserve anonymity. The five geographic regions are British Columbia (BC), the Prairies (Alberta, Saskatchewan and Manitoba), Ontario, Quebec and the Atlantic provinces (New Brunswick, Nova Scotia, Prince Edward Island, and Newfoundland and Labrador). Centers were classified as small if they operated five or fewer treatment units, or large otherwise.

## 3 RESULTS

We summarize the key survey findings below under thematic headings. Complete survey responses are provided in the supplementary material.

### 3.1 Survey participation and center profile

**FIGURE 1:**
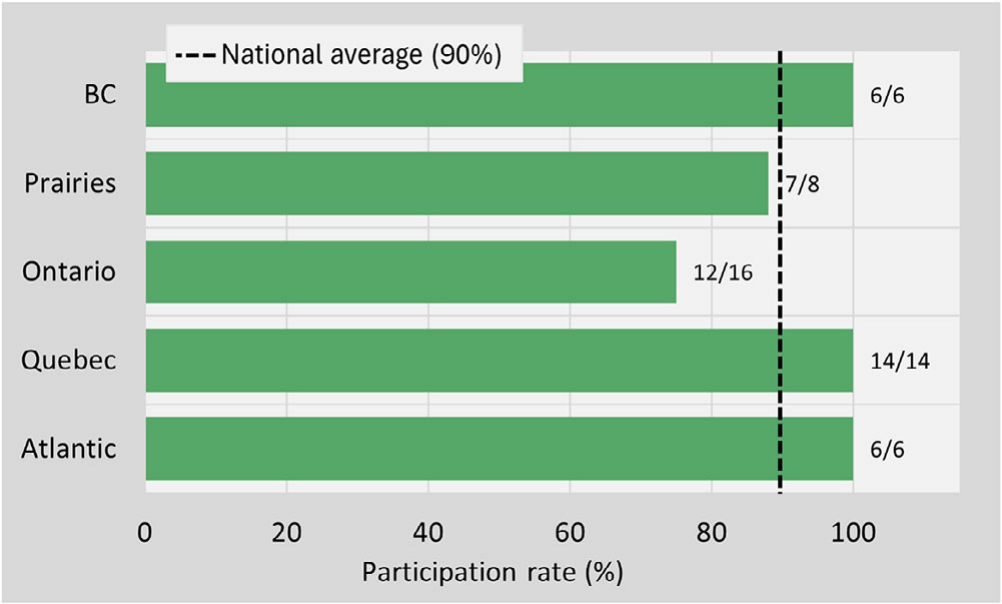
Participation rate by region. The dashed line indicates the national average (90%).

A 90% response rate was achieved from 50 surveyed centers, demonstrating strong engagement. Participation was exceptionally high in BC, Quebec, and the Atlantic, each with a 100% response rate (Figure 1). Among participating centers, 58% employed 10 or fewer FTE physicists, and 62% operated between one and five linacs. Standard C‐arm linacs were the predominant treatment platforms, while a minority of centers utilized specialized machines, including seven with O‐ring linacs, two with MR linacs, and three with CyberKnife units.

### 3.2 PSQA instrumentation and analysis techniques

**FIGURE 2:**
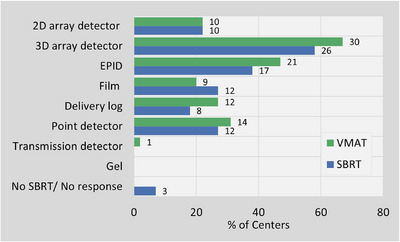
PSQA tools used for VMAT and SBRT plan verification among participating centers.

3D array detectors were identified as the most used PSQA tool across the country, followed by EPID and point detectors for both VMAT and SBRT verification (Figure 2). Although SBRT treatments are frequently delivered using VMAT techniques, they were analyzed separately due to their higher dose per fraction and distinct clinical risk profile. Table 1 further summarizes the measurement setup configurations used with different PSQA detectors for VMAT and SBRT verification.

**TABLE 1:** Percentage of PSQA detectors using true composite (TC), perpendicular field‐by‐field (PFF), and perpendicular composite (PC) measurement setups for VMAT and SBRT verification.

	VMAT			SBRT		
	TC	PFF	PC	TC	PFF	PC
2D array	60%	40%	0%	60%	40%	0%
3D array	97%	0%	3%	96%	0%	4%
EPID	19%	67%	14%	18%	65%	18%
Film	89%	11%	0%	92%	8%	0%
Point	100%	0%	0%	100%	0%	0%

*Note*: Percentages in the SBRT columns for EPID do not sum to 100% due to rounding.

Regional preferences were noted: 3D array detectors were most frequently used in BC and Ontario, whereas EPID was the predominant tool in the Prairies. Quebec showed the greatest diversity in tool selection. Delivery log files were not used for PSQA in the Atlantic, whereas Ontario reported the highest utilization. One center also reported performing in‐vivo transit dosimetry measurements for selected patients.

For VMAT, the distribution of PSQA tool usage across centers was as follows: 29% of centers used only one tool, 38% used two, and 27% used three. This pattern was similar for SBRT. Among centers using only one PSQA tool, the majority selected 3D array detectors, and none used delivery log analysis.

For the analysis, the Gamma index was the primary evaluation method for all PSQA tools except point detectors. For all tools other than delivery log‐based methods, a majority of users also reported utilizing vendor‐provided optional features that can affect pass rates. When assessing dose, a vast majority preferred in‐phantom or in‐detector geometry for PSQA detectors, while in‐patient geometry was favored by the majority of those using delivery log calculations.

### 3.3 Adoption of TG‐218 recommendations

The majority of respondents reported adopting several key recommendations from the AAPM TG‐218 report.^1^ These adopted recommendations included reviewing PSQA outcomes periodically, using a “True Composite” setup for compatible detectors, accounting for machine output variations, employing global normalization in absolute dose, applying a dose difference of 3% and a low‐dose threshold of 10% for Gamma analysis, and setting a Gamma pass rate tolerance level of 95%. The criteria for establishing these parameters were primarily derived from published guidelines, protocols, and in‐house experience.

Relative to the TG‐218 benchmark recommendations, two key metrics—a Gamma analysis using a 2 mm distance‐to‐agreement (DTA) and an action level of 90%—were not consistently adopted by the majority of centers. Most centers performing VMAT PSQA reported using a 3 mm DTA, in contrast to SBRT where most reported using 2 mm. In addition, most EPID users reported applying a higher action level of 95% for both VMAT and SBRT.

### 3.4 Policies, procedures, and failure management

All participating centers reported having official documentation detailing the policies and procedures of their PSQA program, although some noted their documentation was partial rather than complete. Most stored PSQA results both in the patient's official record and in a separate database, while only a few stored results exclusively within PSQA software.

**FIGURE 3:**
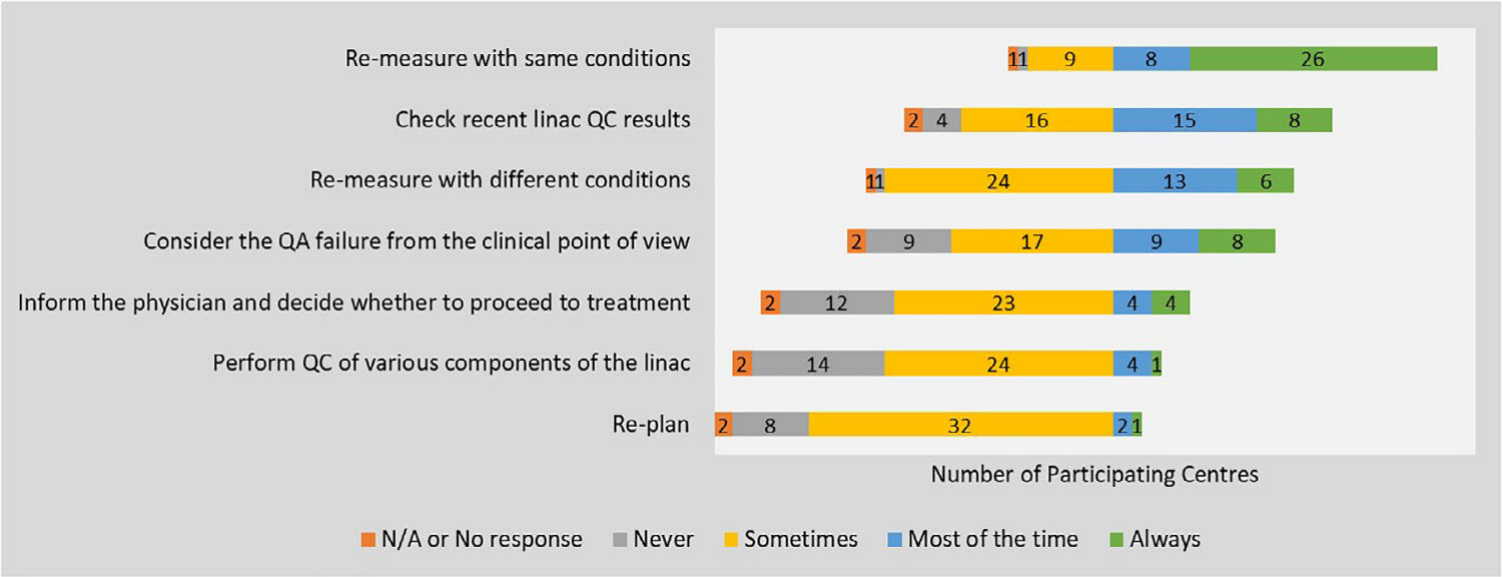
Frequency of actions taken following a failed PSQA measurement result across participating centers.

In the event of a failed PSQA measurement, the most common immediate action was to re‐measure under the same conditions, followed by checking recent linac QC results (type of QC not specified in the survey). The least common action was to re‐plan (Figure 3). For failed delivery log calculations, the primary response was to re‐deliver under the same conditions, followed by performing a direct measurement. The most frequently cited reasons for PSQA failures or suboptimal results were measurement‐related issues and limitations of the beam model. Although the survey did not query specific beam model deficiencies, commonly recognized limitations include MLC tongue‐and‐groove effects, leaf‐end modeling accuracy, small‐field output factors, and mechanical effects such as gantry sag, which can contribute to discrepancies between calculated and delivered dose.

When a PSQA plan failed to meet established criteria, decision‐making responsibility on whether it can proceed to treatment varied across centers. In 51% of centers, the medical physicist was identified as the sole decision‐maker, the physician (24%), either professional (20%), or both professionals jointly (2%). One center (2%) reported that plans failing PSQA criteria were not treated under any circumstances. Percentages do not sum to 100% due to rounding. Additional details are provided in Question 12 of the supplementary material. Across centers that accepted plans failing PSQA criteria, the primary justifications provided were professional judgment and clinical considerations, reflecting a multidisciplinary evaluation process.

A vast majority of centers (82%) required PSQA approval before the first fraction of SBRT, compared with 53% for VMAT. An overwhelming majority of centers (93% VMAT, 84% SBRT) did not perform PSQA for every fraction. The primary reasons given included that per‐fraction PSQA was resource intensive and unnecessary, with some respondents noting reliance on institutional experience and sufficient routine machine QC. Even if the process was efficient, only 22% of respondents would consider performing per‐fraction PSQA.

### 3.5 Adherence to CPQR guidelines and regional variation

**FIGURE 4:**
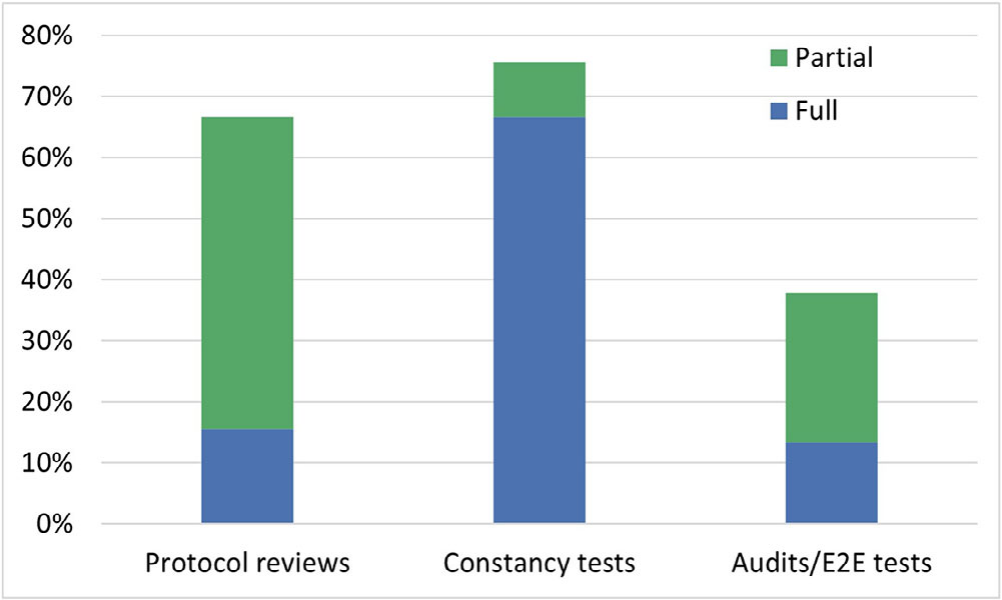
Full and partial compliance of the three CPQR recommended activities.

Adherence was assessed relative to the CPQR technical QC guidance for PSQA, with several areas of partial or non‐adherence identified across the three main recommended activities: quarterly constancy tests, annual protocol reviews, and biennial independent audits or end‐to‐end (E2E) tests (Figure 4).^3^ While quarterly constancy testing showed full adherence by the majority of participating centers, only three centers—all small—reported full adherence to all three recommendations. In total, 29% of centers reported either full or partial adherence to all three CPQR recommended activities. Partial adherence was defined as performing the activity at a reduced frequency compared to the recommended interval.

When examined by center size and region, adherence (full and partial) patterns showed notable variation (Table 2):

- Protocol reviews: 67% conducted protocol reviews, with smaller centers more likely to do so.
- Constancy tests: 76% reported performing constancy tests, with higher compliance among larger centers. In BC, the compliance was 100%.
- Independent audits/E2E tests: Nationwide, 38% of centers reported conducting independent audits/E2E tests. Smaller centers were more likely than larger centers to do so (43% vs 29%), although this pattern varied regionally. For example, large centers reported higher adherence in the Prairies and Quebec.

**TABLE 2:** Percentage of centers reporting adherence (full and partial) to selected CPQR‐recommended PSQA activities, by center size and region.

CPQR practices	Center type	BC	Prairies	Ontario	Quebec	Atlantic	Nation‐wide
Protocol reviews	Small	100%	75%	83%	56%	–	71%
	Large	0%	67%	67%	60%	–	59%
	Overall	67%	71%	75%	57%	67%	67%
Constancy tests	Small	100%	50%	67%	78%	–	68%
	Large	100%	100%	83%	100%	–	88%
	Overall	100%	71%	75%	86%	33%	76%
Independent audits/E2E tests	Small	50%	25%	50%	33%	–	43%
	Large	0%	67%	0%	60%	–	29%
	Overall	33%	43%	25%	43%	50%	38%

*Note*: Centers in the Atlantic region were not categorized by size to protect their anonymity.

These findings provide a national snapshot of adherence to CPQR‐recommended practices. The following section explores how these standards relate to practice patterns, specifically the frequency of PSQA measurements.

### 3.6 PSQA measurement frequency patterns

Across Canada, 53% (*n* = 24) of centers reported reducing the frequency of PSQA measurements for VMAT and/or SBRT, while 47% (*n* = 21) continued performing PSQA for all patient plans. Reported reductions refer to a change relative to each center's initial PSQA frequency.

As illustrated in Table 3, among the measurement frequency‐reduction centers, the most common justification was “performed enough measurements and found no failure” (79%), followed by “based on data‐driven methods” (38%) and “based on plan metrics” (29%). Two centers provided additional context in free‐text comments, citing the use of delivery log analysis and enhanced daily QC. No centers cited published guidelines as a reason for reduction.

**TABLE 3:** Reported justifications among measurement frequency‐reduction centers.

Justification	% of centers
Performed enough measurements and found no failure	79%
Based on data driven methods	38%
Based on plan metrics	29%
Based on risk analysis	13%
We do not have any justification	8%
Based on artificial intelligence/deep learning of plan patterns	4%
Other	4%
Based on published guidelines	0%

#### 3.6.1 Impact of center size and region

Larger centers were more likely than smaller centers to reduce PSQA measurement frequency. Regionally, reductions were reported in BC, Ontario, and Quebec, while all centers in the Prairies and Atlantic reported maintaining full PSQA measurement frequency (Table 4).

**TABLE 4:** Percentage of centers reporting reduced PSQA measurement frequency by center size and region.

Center type	BC	Prairies	Ontario	Quebec	Atlantic	Nationwide
Small	75%	0%	33%	89%	0%	46%
Large	100%	0%	67%	100%	0%	65%
All sizes	83%	0%	50%	93%	0%	53%

#### 3.6.2 Impact of PSQA verification tools

Table 5 summarizes the percentage of centers that reported reducing PSQA measurement frequency, grouped by the verification tool used. For clarity, centers were categorized as: those using delivery log (with or without other tools but not EPID), those using EPID (with or without other tools but not delivery log), and those using neither of these two tools. Centers using the delivery log‐based verification reported the highest rates of reduction for both VMAT (88%) and SBRT (86%), while those using EPID reported much lower rates of reduction (35% VMAT, 40% SBRT). Centers utilizing other tools reported moderate rates of reduction (56% VMAT, 47% SBRT). There were very few centers using both delivery log and EPID, and because this number fell below our grouping threshold of four, these centers were excluded.

**TABLE 5:** Percentage of measurement frequency‐reduction centers by verification tool.

Tools	VMAT	SBRT
Delivery log	88%	86%
EPID	35%	40%
Other	56%	47%

*Note*: Delivery log, delivery log ± other tools but not EPID; EPID, EPID ± other tools but not delivery log; Other, tools not including delivery log or EPID.

#### 3.6.3 Adherence to CPQR‐recommended practices

Among centers that reduced PSQA measurement frequency, full and partial adherence to individual CPQR‐recommended activities is summarized below in Table 6. Given the limited numbers of centers in certain regions, the data are intended for descriptive purposes only and should not be used to draw regional comparisons.

- Protocol reviews: 63% of centers conducted protocol reviews, with similar adherence rate across BC, Ontario, and Quebec.
- Constancy tests: 79% of centers maintained regular constancy testing, ranging from 100% in BC to 50% in Ontario.
- Independent audits/E2E tests: 33% of centers performed independent audits/E2E tests—BC 40%, Ontario 0%, Quebec 46%.

**TABLE 6:** Percentage of centers by region fully and partially adhering to CPQR‐recommended PSQA practices among measurement frequency‐reduction centers.

CPQR practices	BC	Ontario	Quebec	Overall
Protocol reviews	60%	67%	62%	63%
Constancy tests	100%	50%	85%	79%
Independent audits/E2E tests	40%	0%	46%	33%

*Note*: Data are presented descriptively and are not intended for regional comparison.

### 3.7 Program evaluation and future directions

Formal evaluation of PSQA programs was limited. Only 18% of centers performed a risk‐based analysis (e.g., failure mode and effects analysis) of their PSQA program. Table 7 summarizes both current adoption of risk‐based PSQA and centers’ interest in implementing such approaches in the near future.

**TABLE 7:** Adoption of and interest in risk‐based PSQA approaches among participating centers.

Risk analysis performed?	% of centers
Yes	18%
No, but interested	58%
No, not interested	24%

Nationwide, 62% of centers performed sensitivity and specificity characterization of their PSQA system. Interestingly, no measurement frequency‐reduction centers in Ontario performed independent audits/E2E tests, but 83% reported performing PSQA sensitivity and specificity characterization, followed by 60% in BC and 46% in Quebec. Overall, 58% of centers in this subgroup characterized the sensitivity and specificity of their PSQA systems.

When considering PSQA systems, a majority (69%) indicated that vendor independence was either ‘very’ or ‘somewhat’ important. Table 8 summarizes respondents’ perspectives on the importance of QA system independence.

**TABLE 8:** Reported importance of vendor independence between PSQA tools and treatment planning system(s) or the treatment machines among participating centers.

Vendor independence	% of centers
Very important	29%
Somewhat important	40%
Not important	24%
No consensus among staff	7%

Finally, looking forward, there was strong consensus on the need for updated practice guidance, with nearly 90% of centers expressing interest in a Canadian‐centric PSQA guideline.

## 4 DISCUSSION

This national survey provides a snapshot of PSQA practices across Canadian centers, highlighting key patterns, variations, and areas for potential standardization. A key finding of this survey is the uneven adherence to the full suite of CPQR technical QC guidelines.^3^ While most centers reported performing quarterly constancy testing, full adherence with annual protocol reviews and biennial independent audits/E2E tests were less common, indicating that maintaining comprehensive program‐level adherence remains a challenge. Adherence rates to CPQR‐recommended activities were similar among centers that reduced PSQA measurement frequency (Table 6) and all centers nationwide (Table 2), suggesting that guideline adoption was not a principal factor influencing the decision to reduce measurement frequency. Notably, the choice of certain PSQA verification tools appeared to influence practice patterns (Table 5), with centers using delivery log‐based verification reporting markedly higher rates of reduced measurement frequency for both VMAT and SBRT. It is likely that most of these centers substituted many routine patient‐specific measurements with delivery‐log verification. Moreover, since no center reported using the delivery log as its sole tool for PSQA, this reflects a hybrid approach to maintaining PSQA coverage while reducing resource‐intensive measurements. On the other hand, most centers using EPID did not reduce their frequency, likely because EPID dosimetry is less resource‐intensive than the other measurement tools.

The limited use of independent audits/E2E tests warrants attention. Notably, none of the measurement frequency‐reduction centers in Ontario reported performing independent audits or E2E tests, which is unexpected. It is possible that there was a misunderstanding or misreporting of the survey question, or perhaps reliance on alternative mechanisms to ensure confidence in their PSQA processes, such as trial accreditation activities. A study by Kry et al.^9^ reported that 18 of 337 phantom audits failed despite lenient Gamma criteria (7%/4 mm, 85% pass rate), and only one out of these 18 failures was detected by the institutional QA program (6% sensitivity). These findings highlight the value of independent audits in identifying systematic issues that may not be apparent through internal QA alone and suggest that absence of independent external testing may reduce the likelihood of detecting rare but clinically significant errors. This reinforces the value of routine independent audits to complement in‐house QA. This is especially critical for SBRT, where minor delivery errors, such as MLC positional deviations, can cause clinically significant dose errors.^10^, ^11^, ^12^

Another notable finding was the selective adoption of recommendations from the AAPM TG‐218 report. While centers have incorporated most of its key recommendations—including periodic review of PSQA outcomes, accounting for machine output variations, employing global normalization in absolute dose, applying a dose difference of 3% and a low‐dose threshold of 10% for Gamma analysis—most did not adopt the 2 mm DTA criterion for VMAT or the 90% Gamma pass rate action level for EPID. These choices likely reflect pragmatic clinical compromises intended to minimize false positives while maintaining confidence in plan deliverability. TG‐218 guidelines appear to serve as a reference rather than a rigid standard, with centers tailoring parameters based on their specific equipment, clinical experience, and patient populations.

The management of PSQA failures highlights the critical role of medical physicists. Rather than immediately re‐planning, the common response to a failed PSQA is to investigate the measurement process or assess linac performance. The widespread use of professional and clinical judgments to override failed results indicates that PSQA is not viewed as a simple binary pass/fail test. Instead, it serves as one component within a broader decision‐making process, where qualified medical physicists weigh dosimetric errors against potential clinical impact. This experience‐driven approach is also reflected in how PSQA programs evolve: nearly half of all surveyed centers reported no reduction in PSQA measurement frequency, and where reductions occurred, they were primarily driven by extensive institutional experience and data. In fact, the most cited justification for reducing PSQA measurement was that sufficient measurements had been performed without detecting failures (Table 3). While this reflects reliance on local experience, it also underscores the absence of national benchmarks to guide such decisions consistently across centers.

Some limitations of the survey should be acknowledged. Participants were asked to select up to four PSQA tools most utilized at their center. While this restriction helped focus responses on key practices, it may have underrepresented less frequently used tools, potentially failing to capture the full scope of PSQA implementation. Nonetheless, the dominant trends are likely preserved. Notably, several centers selected TC for EPID (Table 1), which is unlikely given that portal imagers are typically gantry mounted, suggesting that some respondents may have misinterpreted their measurement setup configurations. In addition, the survey did not capture absolute PSQA measurement frequencies before and after reported reductions, but rather captured changes relative to each center's baseline practice.

This pan‐Canadian survey identified variation in PSQA practices influenced by center size, regional context, and available resources. Larger centers were more likely to report reduced PSQA measurement frequency, which may indicate confidence in internal processes and accumulated data. This trend, however, coincided with relatively low rates of participation in independent external audits/E2E tests, raising questions about how best to balance internal QA with external oversight. In contrast, smaller centers were more likely to maintain full measurement frequency and to participate in independent audit/E2E testing. Regional differences were also observed, with no centers in the Prairies and Atlantic provinces reporting reductions in PSQA measurement frequency. Together, these findings point to the potential value of a national guidance to promote greater consistency in PSQA practices.

## 5 CONCLUSION

Overall, Canadian centers have implemented thoughtful, experience‐driven PSQA practices that remain variable and locally driven. The findings are presented to describe current practice patterns and are not intended as prescriptive recommendations. However, there is a clear appetite for unified, evidence‐based national standards that are practical, flexible, and scalable across diverse clinical settings, while maintaining treatment quality and patient safety.

## AUTHOR CONTRIBUTIONS

All authors have read the author's professional and ethical responsibilities and authorship requirements on the AAPM website and confirm that they meet the listed criteria.

## FUNDING INFORMATION

The authors received no external funding for this research.

## CONFLICT OF INTEREST STATEMENT

The authors declare no conflicts of interest.

## Supporting information



Supporting Information

## Data Availability

Research data are stored in an institutional repository and will be shared upon request to the corresponding authors.
