# Supplementary material for: COMP Report: Patient‐specific quality assurance practices in Canadian radiotherapy—results from a national survey
Source: J Appl Clin Med Phys. 2026 Apr 21;27(5):e70591. doi: 10.1002/acm2.70591 (PMC13099579; doi:10.1002/acm2.70591)
Supplement: Supplementary file 1 — Supporting Information [file ACM2-27-e70591-s001.docx]

**Supplement: Survey Results**

Note: Results for two survey questions were excluded due to interpretive ambiguity. The first, regarding dose-to-water versus dose-to-medium for PSQA plans (Q16) received inconsistent responses, even among users of the same algorithm or treatment planning system. The second question, on detector dose calibration methods (part of Q17/18), aimed to determine if the detector's reference dose was established via a dosimetry protocol (e.g., TG-51) or a treatment planning system calculation in a reference geometry. However, this question was misinterpreted by some respondents (evidenced by comments on relative/array/MV panel calibration), and many 'Other' responses lacked sufficient detail to determine the reference dose origin, leading to its exclusion.

1. Participating centers in Canada.

| Region | # of Participating Centers | Participation Rate per Region |
| --- | --- | --- |
| BC | 6 | 100% |
| Prairies | 7 | 88% |
| Ontario | 12 | 75% |
| Quebec | 14 | 100% |
| Atlantic | 6 | 100% |

2. How many FTE clinical physicists does your center have?

|  | % of Centers | # of Centers |
| --- | --- | --- |
| 0.1 - 3 | 9% | 4 |
| 3.1 - 6 | 27% | 12 |
| 6.1 - 10 | 22% | 10 |
| **10.1 - 15** | **29%** | **13** |
| 15.1 - 20 | 2% | 1 |
| 20.1 - 25 | 11% | 5 |

3. How many linacs used for patient treatment does your center have?

|  | % of Centers | # of Centers |
| --- | --- | --- |
| **1-5** | **62%** | **28** |
| 6-10 | 31% | 14 |
| 11-15 | 4% | 2 |
| >15 | 2% | 1 |

4. Does your center have official documents detailing the policy and procedures of PSQA, such as measurement or calculation methodology, data preparation in TPS, data analysis and interpretation, and tolerance/action levels?

|  | % of Centers | # of Centers |
| --- | --- | --- |
| **Yes. We have all official documents** | **58%** | **26** |
| Yes. We have some official documents | 42% | 19 |
| No. We do not have any official documents | 0% | 0 |

5. Do the staff at your center review the PSQA protocol, including the process, policy and procedure at least annually as per CPQR Technical Quality Control guidelines for Patient-Specific Dosimetric Measurements for IMRT?

|  | % of Centers | # of Centers |
| --- | --- | --- |
| Yes | 16% | 7 |
| **We conduct the review regularly, but less frequently than annually** | **51%** | **23** |
| No. we do not regularly review the PSQA program | 33% | 15 |

6. Is a PSQA control constancy test (a test plan chosen for repeat delivery) performed for all linacs used to treat VMAT or SBRT at least quarterly as per CPQR Technical Quality Control guidelines for Patient-Specific Dosimetric Measurements for IMRT?

|  | % of Centers | # of Centers |
| --- | --- | --- |
| **Yes** | **67%** | **30** |
| We perform a PSQA constancy test regularly, but less frequently than quarterly | 9% | 4 |
| No. we do not regularly perform a PSQA constancy test | 24% | 11 |

7. Does your center review PSQA results periodically (with the intention of identifying systematic errors in the system)?

|  | % of Centers | # of Centers |
| --- | --- | --- |
| **Yes** | **44%** | **20** |
| Yes, but only for selected techniques/sites/PSQA tools | 20% | 9 |
| No | 36% | 16 |

8. Does your center have an independent audit or review (or complete a full end-to-end test using a phantom or dosimeter that is not employed for your PSQA control tests) at least once every two years as per CPQR Technical Quality Control guidelines for Patient-Specific Dosimetric Measurements for IMRT?

|  | % of Centers | # of Centers |
| --- | --- | --- |
| Yes | 13% | 6 |
| We have regular independent audit or review less frequently than once every two years | 24% | 11 |
| No. we do not have regular independent audit or review | **62%** | **28** |

9. How does your center keep a record of PSQA results (measurements/delivery log calculations)? Select all that apply:

|  | % of Centers | # of Centers |
| --- | --- | --- |
| In the patient's official record (such as Record & Verification system) | **67%** | **30** |
| In a database or spreadsheet | 60% | 27 |
| In the PSQA tool software | 31% | 14 |
| No. we do not keep a record | 0% | 0 |

10. Specific to PSQA *measurement based* techniques, what steps are taken when a plan does not pass PSQA criteria?

|  | % of Centers, # of Centers | | | | | | | | | |
| --- | --- | --- | --- | --- | --- | --- | --- | --- | --- | --- |
|  | N/A or No response | | Never | | Sometimes | | Most of the time | | Always | |
| **Re-measure with same conditions** | **2%** | **1** | **2%** | **1** | **20%** | **9** | **18%** | **8** | **58%** | **26** |
| Check recent linac QC results | 4% | 2 | 9% | 4 | 36% | 16 | 33% | 15 | 18% | 8 |
| Re-measure with different conditions | 2% | 1 | 2% | 1 | 53% | 24 | 29% | 13 | 13% | 6 |
| Consider the QA failure from the clinical point of view | 4% | 2 | 20% | 9 | 38% | 17 | 20% | 9 | 18% | 8 |
| Inform the physician and decide whether to proceed to treatment | 4% | 2 | 27% | 12 | 51% | 23 | 9% | 4 | 9% | 4 |
| Perform QC of various components of the linac | 4% | 2 | 31% | 14 | 53% | 24 | 9% | 4 | 2% | 1 |
| Re-plan | 4% | 2 | 18% | 8 | 71% | 32 | 4% | 2 | 2% | 1 |

11. Specific to PSQA *delivery log calculations*, what steps are taken when a plan does not pass PSQA criteria? [Note that only 11 centers utilized delivery log calculations]

|  | % of Centers, # of Centers | | | | | | | |
| --- | --- | --- | --- | --- | --- | --- | --- | --- |
|  | Never | | Sometimes | | Most of the time | | Always | |
| **Re-deliver with same conditions** | **18%** | **2** | **18%** | **2** | **0%** | **0** | **64%** | **7** |
| Measurement | 18% | 2 | 18% | 2 | 18% | 2 | 45% | 5 |
| Re-deliver with different conditions | 55% | 6 | 27% | 3 | 9% | 1 | 9% | 1 |
| Check recent linac QA results | 55% | 6 | 36% | 4 | 0% | 0 | 9% | 1 |
| Consider the QA failure from the clinical point of view | 73% | 8 | 18% | 2 | 0% | 0 | 9% | 1 |
| Perform QC of various components of the linac | 64% | 7 | 36% | 4 | 0% | 0 | 0% | 0 |
| Re-plan | 82% | 9 | 18% | 2 | 0% | 0 | 0% | 0 |
| Inform the physician and decide whether to proceed to treatment | 91% | 10 | 9% | 1 | 0% | 0 | 0% | 0 |
| Re-calculate delivery log based plan using a different algorithm or beam model | 91% | 10 | 9% | 1 | 0% | 0 | 0% | 0 |

12. While input from all groups may be considered, who ultimately makes the decision on whether plans not meeting PSQA passing criteria should progress to treatment?

|  | % of Centers | # of Centers |
| --- | --- | --- |
| A medical physicist | **51%** | **23** |
| A physician | 24% | 11 |
| Either a medical physicist or a physician | 20% | 9 |
| None. We do not treat plans that fail PSQA criteria | 2% | 1 |
| Both medical physicist and physician together | 2% | 1 |

13. What is the justification for accepting plans for treatment that fail PSQA criteria? Select all that apply:

|  | % of Centers | # of Centers |
| --- | --- | --- |
| Professional judgement | **80%** | **36** |
| Clinical judgement | 67% | 30 |
| Plan score evaluation | 29% | 13 |
| We do not treat plans that fail PSQA criteria | 13% | 6 |
| N/A | 2% | 1 |

14. If your center has reduced the PSQA measurement frequency, what is the justification? Select all that apply:

|  | % of Centers | # of Centers |
| --- | --- | --- |
| No reduction in measurement frequency | **47%** | **21** |
| Performed enough measurements and found no failure | 42% | 19 |
| Based on data driven methods | 20% | 9 |
| Based on plan metrics | 16% | 7 |
| Based on risk analysis | 7% | 3 |
| We do not have any justification | 4% | 2 |
| Based on artificial intelligence/deep learning of plan patterns | 2% | 1 |
| Other | 2% | 1 |
| Based on published guidelines | 0% | 0 |

15. For the following technique, if PSQA is to be performed, does the PSQA have to be approved before the first fraction?

|  | % of Centers, # of Centers | | | |
| --- | --- | --- | --- | --- |
|  | VMAT | | SBRT | |
| **Yes** | **53%** | **24** | **82%** | **37** |
| No | 18% | 8 | 9% | 4 |
| It depends on the number of fractions | 16% | 7 | 0% | 0 |
| It depends on other or multiple factors | 13% | 6 | 4% | 2 |
| N/A. We do not treat with SBRT | N/A | | 4% | 2 |

16. If your treatment planning system allows a choice between dose-to-water and dose-to-medium calculation for your PSQA plans, which one does your center choose?

The responses to the question are withheld due to concerns about data accuracy.

17,18. In these two questions, participants were asked to list up to four most relevant tools commonly utilized for PSQA at their centers for VMAT and SBRT. Then for each tool, participants were asked to select the most appropriate response for each sub-question. The provided tool options were: 2D array detector (e.g. MatriXX, Octavius), 3D array detector (e.g. ArcCHECK, Delta4), EPID, film (e.g. GAFchromic), machine delivery log calculation (e.g. MobiusFX, SunCHECK), point detector (e.g. chamber, diode), transmission detector (e.g. Dolphin, IQM), and gel.

1. PSQA Tools:

|  | % of Centers, # of Centers | | | |
| --- | --- | --- | --- | --- |
|  | VMAT | | SBRT | |
| 2D array detector | 22% | 10 | 22% | 10 |
| **3D array detector** | **67%** | **30** | **58%** | **26** |
| EPID | 47% | 21 | 38% | 17 |
| Film | 20% | 9 | 27% | 12 |
| Machine log calculation | 27% | 12 | 18% | 8 |
| Point detector | 31% | 14 | 27% | 12 |
| Transmission detector | 2% | 1 | 0% | 0 |
| Gel | 0% | 0 | 0% | 0 |
| No SBRT/ No response | 0% | 0 | 7% | 3 |

1. Number of centers that use anthropomorphic phantoms for the detectors**:**

|  | # of Centers | |
| --- | --- | --- |
|  | VMAT | SBRT |
| 2D array detector | 0 | 2 |
| 3D array detector | 1 | 1 |
| Point detector | 1 | 3 |

1. Number of centers that have non-standard linacs and the corresponding PSQA tools used.

|  | VMAT | | SBRT | |
| --- | --- | --- | --- | --- |
|  | # of Centers | PSQA Tools | # of Centers | PSQA Tools |
| Halcyon | 5 | 3D array, EPID, machine log, point detector | 3 | 3D array, EPID, machine log |
| MR linac | 2 | 3D array | 2 | 3D array |
| Cyberknife | 2 | Film, point detector | 3 | Film, point detector |
| Tomotherapy | 2 | 3D array, EPID | 1 | 2D array |

1. Percentage of PSQA detectors using the following setups:

|  | VMAT | | | SBRT | | |
| --- | --- | --- | --- | --- | --- | --- |
|  | True composite | Perpendicular field-by-field | Perpendicular composite | True composite | Perpendicular field-by-field | Perpendicular composite |
| 2D array | **60%** | 40% | 0% | **60%** | 40% | 0% |
| 3D array | **97%** | 0% | 3% | **96%** | 0% | 4% |
| EPID | 19% | **67%** | 14% | 18% | **65%** | 18% |
| Film | **89%** | 11% | 0% | **92%** | 8% | 0% |
| Point | **100%** | 0% | 0% | **100%** | 0% | 0% |

1. Percentage of PSQA tools incorporating output variation in the analysis:

|  | VMAT | | SBRT | |
| --- | --- | --- | --- | --- |
|  | Yes | No | Yes | No |
| 2D array | **60%** | 40% | **50%** | **50%** |
| 3D array | **60%** | 40% | **65%** | 35% |
| EPID | 24% | **76%** | 29% | **71%** |
| Film | **88%** | 13% | **83%** | 17% |
| Machine log | 38% | **63%** | 29% | **71%** |
| Point | **92%** | 8% | **100%** | 0% |

1. Percentage of PSQA tools evaluated in the following geometries:

|  | VMAT | | | SBRT | | |
| --- | --- | --- | --- | --- | --- | --- |
|  | In-phantom/  detector | In-patient | Both in-phantom and in-patient | In-phantom/  detector | In-patient | Both in-phantom and in-patient |
| 2D array | **100%** | 0% | 0% | **100%** | 0% | 0% |
| 3D array | **97%** | 0% | 3% | **96%** | 0% | 4% |
| EPID | **100%** | 0% | 0% | **100%** | 0% | 0% |
| Film | **89%** | 0% | 11% | **92%** | 0% | 8% |
| Machine log | 22% | **78%** | 0% | 17% | **83%** | 0% |
| Point | **93%** | 7% | 0% | **100%** | 0% | 0% |

1. Percentage of PSQA tools evaluated in the following metrics:

|  | VMAT | | | SBRT | | |
| --- | --- | --- | --- | --- | --- | --- |
|  | Gamma Alone | Gamma and other metrics | Other metrics | Gamma Alone | Gamma and other metrics | Other metrics |
| 2D array | **60%** | 40% | 0% | **60%** | 30% | 10% |
| 3D array | **57%** | 40% | 3% | **62%** | 35% | 4% |
| EPID | **52%** | 43% | 5% | **65%** | 29% | 6% |
| Film | **56%** | 33% | 11% | **67%** | 33% | 0% |
| Machine log | **42%** | 42% | 17% | **50%** | 38% | 13% |
| Point | 7% | 0% | **93%** | 0% | 0% | **100%** |

1. Percentage of PSQA tools using the following dose difference % values in Gamma analysis:

|  | VMAT | | | | SBRT | | | |
| --- | --- | --- | --- | --- | --- | --- | --- | --- |
|  | 2% | 3% | 5% | Other | 2% | 3% | 5% | Other |
| 2D array | **50%** | **50%** | 0% | 0% | 40% | **60%** | 0% | 0% |
| 3D array | 20% | **73%** | 3% | 3% | 23% | **69%** | 4% | 4% |
| EPID | 10% | **86%** | 0% | 5% | 29% | **65%** | 0% | 6% |
| Film | 22% | **78%** | 0% | 0% | 8% | **75%** | 17% | 0% |
| Machine log | 10% | **70%** | 20% | 0% | 14% | **71%** | 14% | 0% |

1. Percentage of PSQA tools using the following distance-to-agreement values in Gamma analysis:

|  | VMAT | | | | SBRT | | | |
| --- | --- | --- | --- | --- | --- | --- | --- | --- |
|  | 1mm | 2mm | 3mm | Other | 1mm | 2mm | 3mm | Other |
| 2D array | 10% | **50%** | 40% | 0% | 10% | **60%** | 30% | 0% |
| 3D array | 0% | **57%** | 40% | 3% | 4% | **73%** | 19% | 4% |
| EPID | 0% | 29% | **67%** | 5% | 12% | **47%** | 35% | 6% |
| Film | 0% | 22% | **78%** | 0% | 17% | **42%** | **42%** | 0% |
| Machine log | 10% | 30% | **60%** | 0% | 14% | 29% | **57%** | 0% |

1. Percentage of PSQA tools using the following low dose threshold % values in Gamma analysis:

|  | VMAT | | | | | SBRT | | | | |
| --- | --- | --- | --- | --- | --- | --- | --- | --- | --- | --- |
|  | 5% | 10% | 15%, 20% | 30%, 40% | N/A or Other | 5% | 10% | 15%, 20% | 30%, 40% | N/A or Other |
| 2D array | 20% | **50%** | 30% | 0% | 0% | 10% | **70%** | 10% | 0% | 10% |
| 3D array | 7% | **70%** | 13% | 7% | 3% | 4% | **73%** | 12% | 8% | 4% |
| EPID | 10% | **71%** | 0% | 5% | 14% | 6% | **76%** | 0% | 6% | 12% |
| Film | 0% | **38%** | **38%** | 13% | 13% | 8% | **58%** | 25% | 8% | 0% |
| Machine log | 11% | **67%** | 0% | 0% | 22% | 14% | **71%** | 0% | 0% | 14% |

1. Percentage of PSQA tools having the following Gamma pass rate tolerance limits:

|  | VMAT | | | | | | SBRT | | | | |
| --- | --- | --- | --- | --- | --- | --- | --- | --- | --- | --- | --- |
|  | 90% | >90%, <95% | | 95% | >95% | N/A or Other | 90% | >90%, <95% | 95% | >95% | N/A or Other |
| 2D array | 10% | 0% | **70%** | | 20% | 0% | 10% | 0% | **90%** | 0% | 0% |
| 3D array | 7% | 0% | **80%** | | 7% | 7% | 8% | 0% | **81%** | 0% | 12% |
| EPID | 5% | 0% | **81%** | | 10% | 5% | 12% | 0% | **71%** | 6% | 12% |
| Film | 25% | 13% | **63%** | | 0% | 0% | 25% | 0% | **58%** | 8% | 8% |
| Machine log | 27% | 0% | **45%** | | 18% | 9% | 29% | 0% | **57%** | 14% | 0% |

1. Percentage of PSQA tools having the following Gamma pass rate action limits:

|  | VMAT | | | | | | SBRT | | | | | |
| --- | --- | --- | --- | --- | --- | --- | --- | --- | --- | --- | --- | --- |
|  | 85% | 90% | >90%, <95% | 95% | >95% | N/A or Other | 85% | 90% | >90%, <95% | 95% | >95% | N/A or Other |
| 2D array | 11% | **33%** | 0% | **33%** | 22% | 0% | 10% | **50%** | 10% | 30% | 0% | 0% |
| 3D array | 7% | **55%** | 0% | 28% | 0% | 10% | 8% | **56%** | 0% | 24% | 0% | 12% |
| EPID | 0% | 25% | 5% | **60%** | 5% | 5% | 0% | 31% | 6% | **50%** | 6% | 6% |
| Film | 25% | **50%** | 0% | 13% | 0% | 13% | 18% | **45%** | 0% | 27% | 0% | 9% |
| Machine log | 20% | **50%** | 10% | 10% | 0% | 10% | 17% | **67%** | 0% | 17% | 0% | 0% |

1. Percentage of PSQA tools using vendor’s optional features that can change pass rates (e.g. auto-shift, measurement uncertainty, and dose scaling):

|  | VMAT | | | SBRT | | |
| --- | --- | --- | --- | --- | --- | --- |
|  | Yes, routinely | Yes, but at user's discretion | No | Yes, routinely | Yes, but at user's discretion | No |
| 2D array | 30% | 30% | **40%** | 20% | **50%** | 30% |
| 3D array | 13% | **53%** | 33% | 12% | **50%** | 38% |
| EPID | **38%** | 29% | 33% | 35% | 24% | **41%** |
| Film | 11% | **56%** | 33% | 18% | **55%** | 27% |
| Machine log | 0% | 0% | **100%** | 0% | 0% | **100%** |

1. Percentage of PSQA tools using the following dose normalization methods:

|  | VMAT | | | SBRT | | |
| --- | --- | --- | --- | --- | --- | --- |
|  | Global normalization in absolute dose | Local normalization in absolute dose | Other | Global normalization in absolute dose | Local normalization in absolute dose | Other |
| 2D array | **78%** | 22% | 0% | **90%** | 10% | 0% |
| 3D array | **83%** | 17% | 0% | **84%** | 16% | 0% |
| EPID | **80%** | 5% | 15% | **81%** | 6% | 13% |
| Film | **89%** | 11% | 0% | **83%** | 17% | 0% |
| Machine log | **60%** | 10% | 30% | **83%** | 0% | 17% |

19. If your center evaluates Gamma in the PSQA analysis, do you examine the pass rate of individual treatment fields (arcs), or the whole plan (composite of all fields)?

|  | % of Centers | # of Centers |
| --- | --- | --- |
| **Whole plan** | **47%** | **21** |
| Both individual fields and whole plan | 38% | 17 |
| Individual fields/arcs | 16% | 7 |

20. For PSQA Gamma analysis, please specify how your center established the following:

a) Evaluation criteria for DD, DTA, and low dose threshold. Select all that apply:

|  | % of Centers | # of Centers |
| --- | --- | --- |
| Followed on published guidelines and protocols | **84%** | **38** |
| Based on in-house experience | 76% | 34 |
| Followed other centers in the medical physics community | 42% | 19 |
| Other | 2% | 1 |

b) Tolerance and action levels. Select all that apply:

|  | % of Centers | # of Centers |
| --- | --- | --- |
| Based on in-house experience | **78%** | **35** |
| Followed on published guidelines and protocols | 76% | 34 |
| Followed other centers in the medical physics community | 36% | 16 |
| Based on statistical process control/analysis | 13% | 6 |
| Other | 2% | 1 |

21. Indicate how often an investigation has led to the following factors as the reason for PSQA failures or suboptimal results at your center.

|  | % of Centers, # of Centers | | | | | | | | | | | |
| --- | --- | --- | --- | --- | --- | --- | --- | --- | --- | --- | --- | --- |
|  | N/A or No response | | 0% (Never) | | 1%-25% | | 26%-50% | | 51%-75% | | 76%-100% | |
| **Measurement related** | **16%** | **7** | **2%** | **1** | **49%** | **22** | **16%** | **7** | **7%** | **3** | **11%** | **5** |
| Beam model limitation | 20% | 9 | 9% | 4 | 44% | 20 | 11% | 5 | 7% | 3 | 9% | 4 |
| Error in analysis | 22% | 10 | 13% | 6 | 47% | 21 | 11% | 5 | 7% | 3 | 0% | 0 |
| Undetermined | 24% | 11 | 20% | 9 | 49% | 22 | 4% | 2 | 2% | 1 | 0% | 0 |
| Linac suboptimal calibration | 22% | 10 | 51% | 23 | 27% | 12 | 0% | 0 | 0% | 0 | 0% | 0 |
| Plan integrity related | 20% | 9 | 67% | 30 | 13% | 6 | 0% | 0 | 0% | 0 | 0% | 0 |

22. For the following technique, does your center currently perform PSQA (measurement or delivery log) for every single fraction?

|  | % of Centers, # of Centers | | | |
| --- | --- | --- | --- | --- |
|  | VMAT | | SBRT | |
| Yes | 7% | 3 | 9% | 4 |
| Yes, for some plans | 0% | 0 | 2% | 1 |
| **No** | **93%** | **42** | **84%** | **38** |
| N/A. We do not treat with SBRT | N/A | | 4% | 2 |

23. If PSQA is not performed for every fraction at your center, why? Select all that apply:

|  | % of Centers | # of Centers |
| --- | --- | --- |
| Resource intensive | **76%** | **34** |
| Deemed unnecessary | 69% | 31 |
| No proper tools | 36% | 16 |
| We performed PSQA for every fraction | 7% | 3 |
| Other | 4% | 2 |

24. Dosimetry using EPID, transmission detector, or delivery log calculations provide an efficient way of performing PSQA. Would the majority of the physicists want PSQA performed for every fraction if your center has these tools?

|  | % of Centers | # of Centers |
| --- | --- | --- |
| Yes | 22% | 10 |
| **No** | **44%** | **20** |
| Undecided | 33% | 15 |

25. Does your center currently perform in-vivo transit (with patient in the beam) dosimetry measurements (typically with an EPID)?

|  | % of Centers | # of Centers |
| --- | --- | --- |
| No intention to perform in the near future | **71%** | **32** |
| No, but would like to in the next three years | 27% | 12 |
| Yes, for some patients | 2% | 1 |
| Yes, for most or all patients | 0% | 0 |

26. Has your center characterized the sensitivity and specificity of your PSQA system?

|  | % of Centers | # of Centers |
| --- | --- | --- |
| Yes, fully | 9% | 4 |
| Yes, partly | **53%** | **24** |
| No | 36% | 16 |
| Other: Do not know | 2% | 1 |

27. Would you like to see a Canadian centric guidance document for PSQA practice?

|  | % of Centers | # of Centers |
| --- | --- | --- |
| **Yes** | **89%** | **40** |
| No | 9% | 4 |
| No response | 2% | 1 |

28. How important is it for your center to have vendor independence between your PSQA tools and the treatment planning system(s) or the treatment machines?

|  | % of Centers | # of Centers |
| --- | --- | --- |
| Very important | 29% | 13 |
| Somewhat important | **40%** | **18** |
| Not important | 24% | 11 |
| The staff are more or less evenly split in the opinion | 7% | 3 |

29. Has your center performed a risk-based analysis of your PSQA program?

|  | % of Centers | # of Centers |
| --- | --- | --- |
| Yes | 18% | 8 |
| **No, but would like to in the next three years** | **58%** | **26** |
| No, and no intention to perform in the near future | 24% | 11 |

30. Please enter any additional comment you may have:

Selected comments:

- Some of the wording was difficult to interpret and may have impacted answers. Practice same for VMAT and SBRT. SRS we do have different practice for and this was not captured in the survey.
- Thank you for putting this together, looking forward to understanding more about the national landscape (particularly around per-fraction monitoring).
- Thanks for doing this. This is a very important aspect of our role in quality patient care. Like I always say to the residents: if an RO makes a mistake, it impacts one patient. If medical physicists make a mistake, it impacts 100's or even 1000’s.
- Thanks for coordinating this. Looking forward to seeing the results.
- Table 18 is very long to fill, ideally a check box that says: answers identical to table 17...
- Faudrait pas que les AQSP faits avec les journaux de livraison soient les seuls effectués. Faut aussi un minimum d'échantillonnage de cas faits avec de vraies mesures.
